# Chondroitin 6-sulphate is required for neuroplasticity and memory in ageing

**DOI:** 10.1038/s41380-021-01208-9

**Published:** 2021-07-16

**Authors:** Sujeong Yang, Sylvain Gigout, Angelo Molinaro, Yuko Naito-Matsui, Sam Hilton, Simona Foscarin, Bart Nieuwenhuis, Chin Lik Tan, Joost Verhaagen, Tommaso Pizzorusso, Lisa M. Saksida, Timothy M. Bussey, Hiroshi Kitagawa, Jessica C. F. Kwok, James W. Fawcett

**Affiliations:** 1 John van Geest Centre for Brain Repair, University of Cambridge, Cambridge, UK; 2 School of Biomedical Sciences, Faculty of Biological Sciences, University of Leeds, Leeds, UK; 3 Institute of Neuroscience, CNR, Pisa, Italy; 4 Department of Biochemistry, Kobe Pharmaceutical University, Kobe, Japan; 5 Laboratory for Regeneration of Sensorimotor Systems, Netherlands Institute for Neuroscience, Royal Netherlands Academy of Arts and Sciences (KNAW), Amsterdam, The Netherlands; 6 Division of Neurosurgery, National University Hospital, Singapore, Singapore; 7 Department NEUROFARBA, University of Florence, Florence, Italy; 8 Molecular Medicine Research Group, Robarts Research Institute, Schulich School of Medicine and Dentistry, Western University, London, Canada; 9 Centre for Reconstructive Neuroscience, Institute of Experimental Medicine CAS, Prague, Czech Republic

**Keywords:** Neuroscience, Biochemistry

## Abstract

Perineuronal nets (PNNs) are chondroitin sulphate proteoglycan-containing structures on the neuronal surface that have been implicated in the control of neuroplasticity and memory. Age-related reduction of chondroitin 6-sulphates (C6S) leads to PNNs becoming more inhibitory. Here, we investigated whether manipulation of the chondroitin sulphate (CS) composition of the PNNs could restore neuroplasticity and alleviate memory deficits in aged mice. We first confirmed that aged mice (20-months) showed memory and plasticity deficits. They were able to retain or regain their cognitive ability when CSs were digested or PNNs were attenuated. We then explored the role of C6S in memory and neuroplasticity. Transgenic deletion of chondroitin 6-sulfotransferase (*chst3*) led to a reduction of permissive C6S, simulating aged brains. These animals showed very early memory loss at 11 weeks old. Importantly, restoring C6S levels in aged animals rescued the memory deficits and restored cortical long-term potentiation, suggesting a strategy to improve age-related memory impairment.

## Introduction

Perineuronal nets (PNNs) are condensed extracellular matrix structures that surround the soma and proximal dendrites of some classes of neurons [1]. In the brain, these are particularly GABAergic parvalbumin^+^ (PV^+^) interneurons. PNNs surround synapses and are involved in the control of developmental and adult plasticity [2–6]. PNNs are formed via hierarchical assembly of various chondroitin sulphate proteoglycans (CSPGs), hyaluronan, tenascin-R, hyaluronan and proteoglycan link proteins (haplns) and other PNN-associated extracellular matrix (ECM) molecules such as semaphorin 3 A [7–11]. Their ability to control plasticity depends on the sulphated chondroitin sulphate glycosaminoglycan chains (CS-GAGs) of the CSPGs [12, 13].

The biochemical functions and binding properties of CS-GAGs are heavily determined by their pattern of sulphation [11, 14–17]. In the central nervous system (CNS), the CS composition is dominated by two mono-sulphated CSs, C6S and C4S, with a minority of di-sulphated CSs [18, 19]. While C6S is permissive to axon growth and plasticity, C4S is inhibitory [12, 13]. The sulphation composition of CS changes with age, with a late decline in permissive C6S. C6S constitutes 18% of the CS-GAGs at birth, declines to 4% at the end of critical periods of development, and further to <1% in aged rats [15, 18, 19]. This decrease leads to an increase in the ratio of C4S/C6S and results in the PNN matrix becoming more inhibitory in aged brains. This change has the potential to further diminish plasticity in the aged CNS [19].

Recently evidence has emerged that PNNs play an important part in memory. Fear memory, drug addiction memory, place memory, associative motor learning and object recognition memory are all modulated by PNN function, and attenuation of PNNs through chondroitinase ABC (ChABC) digestion and transgenic deletion of PNN components can affect memory acquisition, erasure and persistence [20–23]. In spatial, associative motor and object memory, PNN-bearing PV^+^ neurons are implicated, and during memory acquisition new inhibitory inputs are formed on these neurons [23–26]. In young animals, digestion of inhibitory CS-GAG chains (the main effectors of PNNs) with ChABC enables the increased formation of inhibitory synapses on PV^+^ neurons [26, 27], and enhances memory acquisition and duration in rodents and similar effects are seen on deep cerebellar nucleus neurons during associative motor learning [23]. A similar observation of memory enhancement is also found in mice with transgenic attenuation of PNNs [22, 28]. In animal models of Alzheimer’s disease in which animals develop pathology from mutant tau or amyloid-beta, ChABC digestion or CS-GAG blocking with using a C4S-blocking antibody have also alleviated the pathology-associated memory loss [29–31]. Together these results demonstrate a role for PNNs in memory and memory restoration and show that PNN CS-GAGs are a key component in these processes [32, 33].

PNNs become more inhibitory with ageing due to the changes in sulphation of CS-GAGs described above. For the formation of new synapses onto PV^+^ neurons, neuronal processes must penetrate the PNNs. As these structures become more inhibitory with age, synaptogenesis that underlies the formation of new memories may therefore be partially blocked [1]. We hypothesised that the age-related increase in the ratio of C4S/C6S may make PNNs more inhibitory, leading in turn to memory loss associated with diminished inhibitory synapse formation onto PV^+^ interneurons. Changing PNN sulphation may therefore be a contributor to age-related memory impairment (ARMI). We have tested this hypothesis by changing the sulphation pattern of PNNs in young and aged animals and measuring memory performance, using memory tests that can be achieved by very aged animals. Our results show that aged mice develop memory impairment in SOR, SA and marble burying (MB) tasks, and show a concurrent decline in the permissive C6S in PNNs. The memory impairment was restored by digesting CS-GAGs in the brain with ChABC, and transgenic animals with attenuation of PNNs showed no ARMI. Manipulation of the C6S levels in the brain induced changes in memory. Lowered C6S led to premature memory loss but enhanced C6S prevented ARMI and restored memory in established ARMI. Restoration of plasticity was shown through C6S-induced restoration of long term potentiation (LTP) in the aged hippocampus and cortex, and corresponding changes in the number of inhibitory synapses on PV^+^ interneurons. The changing properties of PNNs with age are therefore a factor in ARMI, and targeting sulphation in PNNs could be an effective means of treating and preventing ARMI.

## Results

### Aged mice show memory deficits in SOR, SA and MB tasks

We first established that aged 20 month-old (20 M) mice have a progressive memory and cognitive deficit compared to young 6 month-old mice (6 M) using three tests (a) spontaneous object recognition (SOR), (b) spontaneous alternation (SA), and (c) MB. Unlike young and middle-aged mice that can perform consistently in common memory tasks, such as Morris water maze and Barnes maze, aged mice struggle to stay afloat and swim in water mazes and participate marginally in Barnes mazes [34]. The use of SOR, SA and MB allows for the assessments of function originating from different brain regions, including the perirhinal cortex (PRh) for SOR, the hippocampus for SA and the frontal/prefrontal cortex for MB [35]. SOR and MB are assays that depend on the natural exploratory response of rodents to a novel environment while SA reflects place memory [35, 36]. The tasks show deficits after lesions, and SOR is used as a sensitive measure of disability in Alzheimer models [30, 37].

**Fig. 1:**
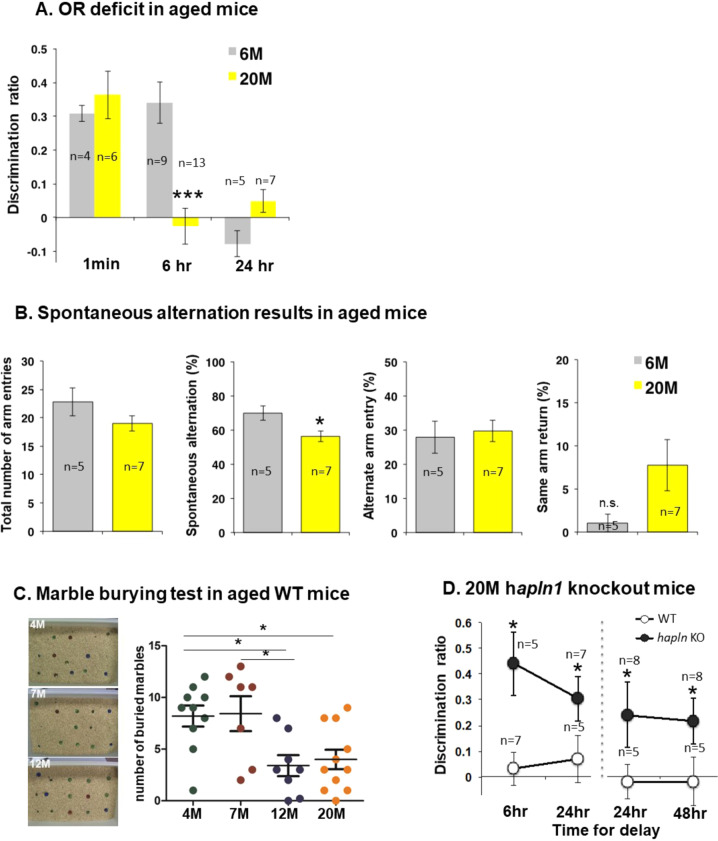
Memory loss in aged mice, rescued by perineuronal net attenuation. **A** OR memory deficit in aged mice. Time of delay 1 min, 6 h, 24 h. Unpaired two-tailed *t*-test, ***p = 0.0002. **B** Spontaneous alternation deficit in aged mice. A total number of arm entries 6 M vs 20 M, 22.8 ± 2.4 vs 19 ± 1.38. Spontaneous alternation (%) 69.9 ± 4.3 vs 56.3 ± 3.1, unpaired two-tailed *t*-test, *p = 0.0392, alternate arm entry (%) 27.9 ± 4.6 vs 29.7 ± 3.1. Same arm return (%) 1.05 ± 1.05 vs 7.76 ± 2.98. **C** Marble burying test in C57bl/6 at different ages, left: representative images of marble-burying test right: 4 M *n* = 10, 7 M *n* = 7, 20 M *n* = 11, One-way ANOVA **p = 0.004, Tukey post hoc test, 4 M vs 12 M *p < 0.05, 4 M vs 20 M *p < 0.05, 7 M vs 12 M *p < 0.05, Data represents as mean ± SEM. **D** SOR memory in aged *hapln1* KO mice (two separate experiments). Unpaired two-tailed *t*-test. 6 h delay: *p = 0.0094. Twenty-four hours: *p = 0.032, *p = 0.0226. Forty-eight hours delay: *p = 0.0145.

At 20 M, C57BL/6 mice showed defective retention of SOR: normally young animals show memory of the novelty of objects at 6 h (h) after exposure to the objects, forgetting by 24 h (Fig. 1A). In aged 20 M animals, SOR memory was barely detectable at 6 h while 6 M animals showed robust memory. However, aged animals were able to form short-term memories similarly to young animals, detected by testing the memory within a minute of object exposure (Fig. 1A). Participation in the tasks remained normal (Fig. S1A) indicating cognitive decline rather than a motor or attention deficit. SA was normal in 6 M mice, but at 20 M an abnormally high rate of same arm re-entry and reduced SAs were observed (Fig. 1B). In the MB test, mice behaved normally at 4 and 7 M, but at 12 and 20 M, they buried abnormally few marbles (Fig. 1C).

### Transgenic attenuation of PNNs prevents ARMI

We have previously shown that PNN removal increases memory retention in young adult mice and restores memory in tauopathy mice [22, 31]. In order to investigate whether memory loss in ageing is also related to PNNs, we assessed memory loss using SOR in transgenic animals with attenuated PNNs. One of the key molecules involved in PNN formation is “hyaluronan and proteoglycan link protein 1” (*hapln1)* which is necessary for the stable binding of CSPGs to hyaluronan. Transgenic deletion of *hapln1* leads to marked attenuation of PNNs, but brains contain normal quantities of CSPGs which are now diffusely spread [7, 38]. In previous work, we have shown that SOR memory is prolonged and various forms of plasticity are enhanced in non-aged *hapln1* knockout mice [22, 38]. To investigate the role of PNNs in memory loss in ageing, we tested SOR in *hapln1* knockout animals at time points from 12 M up to 20 M. While control WT littermates showed clear memory deficits at 20 M with loss of memory at 6 h after object exposure, *hapln1* knockout mice showed no loss of SOR memory, with memory retention extended to 48 h (Fig. 1D). This result implicates PNNs in the loss of SOR memory in aged mice.

### Chondroitinase digestion of CS-GAGs restores memory

**Fig. 2:**
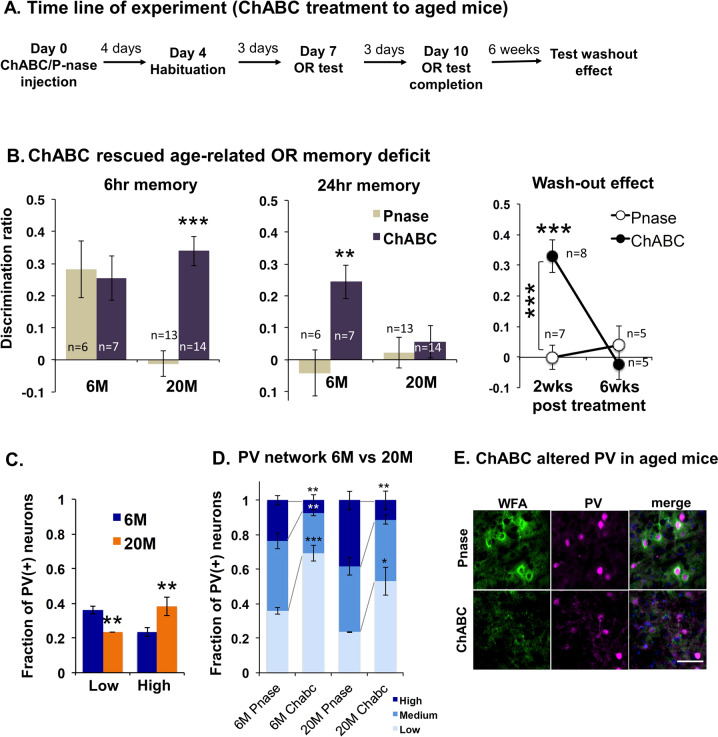
Chondroitinase ABC treatment restores memory and Parvalbumin levels. **A** Timeline of ChABC treatment to young and aged mice. **B** ChABC injection rescued age-related OR memory deficit. 6 h memory at 20 M, unpaired two-tailed *t*-test, ***p = 0.0001. Twenty-four hours memory at 6 M, unpaired two-tailed *t*-test, **p = 0.0074. Wash-out of ChABC treatment effect of 6 h memory in 20 M mice. 2-Way ANOVA interaction treatment × time, ***p < 0.001. Main effect of Time: **p < 0.01. Treatment: **p < 0.01. Bonferroni posthoc test, ChABC 2 weeks vs 6 weeks, ***p < 0.001. Two weeks Pnase vs ChABC, ***p = 0.0002. **C** PV network in aged mice. 6 M vs 20 M low PV: **p = 0.0012, high PV **p = 0.0061, *n* = 6/group. Data present as mean ± SEM. **D** PV network modification after ChABC treatment in 6 M vs 20 M old mice 6 M Pnase vs ChABC *n* = 6/group, low: ***p < 0.0001 medium: **p = 0.0027 high: **p = 0.0028, 20 M Pnase vs ChABC *n* = 4/group, low: *p = 0.019 High: **p = 0.0099. **E** Representative images of the staining profiles of WFA (green), PV (magenta) and DAPI (blue) in 20 M old mice after Pnase and ChABC treatment. Scale bar: 50 µm.

The effects of PNNs are dependent on the content of CS-GAGs [5]. To investigate a link between CSPGs and memory loss in the aged brain, we used ChABC to digest CS-GAGs in the PRh (which regulates SOR memory). ChABC was injected focally into the PRh. The effect of a single ChABC treatment lasts for at least 10 days [39], giving time to assay for the effects of CS-GAGs on memory as in previous experiments [22]. ChABC completely digests both C4S and C6S (Fig. S2) [40]. An injection of ChABC into the PRh of 20 M mice completely restored 6 h memory retention, to a level similar to that seen in 6 M adult mice. Extended memory to 24 h after object exposure was seen in young adult mice treated with ChABC but not in aged mice (Fig. 2B). Following this restoration of memory, the memory declined again as the digested CSPGs were re-synthesised. By 6 weeks after treatment memory was again defective and back to baseline in the 20 M mice (timeline Fig. 2A, B). SA and MB (which do not depend on PRh) are controlled by brain regions remote from the injection sites and are therefore not affected by this focal treatment and remained impaired. The results indicate that CS-GAGs in the PRh are responsible for memory loss in the aged mice.

In young animals increased numbers of inhibitory synapses onto PV^+^ interneurons are seen during memory acquisition, leading to decreased PV [26]. ChABC-mediated CS-GAG digestion in young animals similarly decreases PV expression, indicative of changes in GABAergic inhibition [26, 41]. We counted PV^+^ neurons expressing high, medium and low levels of PV as in these previous studies. At 20 M we saw an increase in the proportion of high-expressing PV^+^ cells and a decrease in low-expressing PV^+^ neurons in the aged cortex compared to 6 M (Fig. 2C). ChABC treatment at both ages increased low-expressing and decreased high-expressing PV^+^ neurons (Figs. 2D, E and S1D). The overall number of neurons and PNNs in PRh was unchanged during ageing (Fig. S1B). Overall, these experiments show that SOR memory in aged animals can be restored by attenuation of PNNs or digestion of CS-GAGs in PRh. Ageing is associated with increased PV expression in PV^+^ interneurons, levels being returned to those of young animals by ChABC treatment. Together with the results from the link protein (*hapln 1*) knockouts, the conclusion is that the digestion of CS-GAGs or attenuation of PNNs can restore memory function in ARMI.

### Transgenic reduction of C6S leads to premature memory loss

**Fig. 3:**
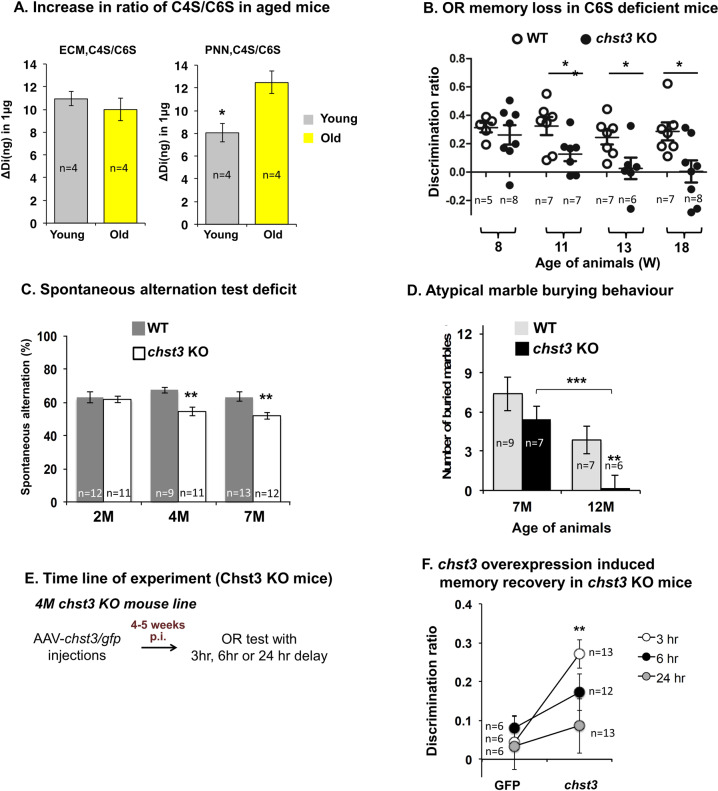
Aged mice lose C6S. Knockout of C6 sulfotransferase levels lead to premature memory loss. **A** Increase in the ratio of C4S/C6S in aged mice suggests a depletion of C6S. Left, general interstitial matrix. Right, PNN matrix. **B** OR memory loss (3 h) in *chst3* knockout mice. Unpaired two-tailed *t*-test. 11 weeks: *p = 0.0317. 13 weeks: *p = 0.0306. 18 weeks: *p = 0.0165. **C** Impaired spontaneous alternation performance in *chst3* knockout mice. Unpaired two-tailed *t*-test, 4 M: **p = 0.0011. 7 M: **p = 0.001. **D** Atypical marble-burying behaviour in *chst3* KO mice on ageing. Unpaired two-tailed *t*-test. 12 M: **p < 0.01. In WT the ageing impaired marble-burying behaviour, 7 M vs 12 M ***p < 0.001. **E** Experimental timeline of AAV-*chst3* injection to *chst3* KO mice at 4 M. **F** *chst3* gene delivery recovered memory in 4 M *chst3* KO mice line. Unpaired two-tailed *t*-test, 3 h delay: **p < 0.01.

Our hypothesis was that the loss of the more permissive C6S, causing an increased ratio of C4S/C6S in the aged brain, leads to CSPGs becoming increasingly inhibitory, worsening memory deficits in the aged brain. Most of the functions of GAGs reside in the sulphation composition of the GAG chain [12, 13, 17], we, therefore, investigated the age-related changes in the CS-GAGs from the PNNs. We isolated GAGs from the interstitial ECM and PNNs of 6 M and 20 M mouse brains and quantified the C4S and C6S content. We found a reduction of C6S in the PNNs of 20 M mice, leading to an increase in the ratio of C4S to C6S by 56% (Fig. 3A). This age-related sulphation change was not observed in the interstitial ECM. The mouse results were very similar to rats [19] suggesting that the down-regulation of the more permission C6S in PNNs occurs generally in aged rodents. Evidence that the balance between C6S and C4S determines inhibition of neurite growth on brain ECM is shown in Fig. S3. ECM from animals lacking C6S showed strong inhibition of neurite outgrowth because inhibitory C4S is now dominant. Enzymatic removal of the 4-sulphation to convert C4S to non-sulphated C0S made the ECM more permissive although C6S is still present.

If loss of C6S is a causal factor in ARMI, a decrease in C6S at any age would be expected to lead to memory impairment. To test whether premature C6S loss induces early memory deficits, SOR, SA and MB were tested in young C6 sulfotransferase-1 (*chst3*) knockout animals. These mice showed a marked reduction of C6S while C4S levels were normal, and the ECM was abnormally inhibitory [12]. The absence of 6-sulphated CS in these animals is shown in Fig. S4 and the length of WFA-stained PNNs along the dendrites of PV^+^ interneurons did not differ between controls and *chst3* knockouts (Fig. S5). While the WT littermate mice demonstrated normal SOR memory formation and retention between 8 and 18 weeks of age (W), *chst3* knockout mice demonstrated progressive premature memory deficits from 11 W. By 13 W there was no measurable memory retention even at 3 h after exposure to the objects (Fig. 3B). These animals also showed similar premature deficits in spatial memory as measured by SA. By 4 M of age, the knockout animals showed an increased frequency of immediate re-visiting the same arm of the maze (Figs. 3C and S6B). The animals also showed defective marble-burying at 12 M (Fig. 3D). Immunohistochemistry in the retrosplenial and perirhinal cortices show that the number of PNNs in these regions was normal as indicated by WFA staining (Fig. S6C). This suggests that premature memory deficits in these mice are due to the change in the biochemical nature of the PNNs but not the overall number of neurons bearing PNNs.

In order to confirm that the loss of memory was due to changes in C6S levels and not due to an idiosyncrasy of the transgenic, we tested whether restoration of C6S could recover memory in *chst3* knockout mice using SOR. An AAV1 vector expressing mouse *chst3* under a PGK promoter was injected into the PRh at 4 months of age (Fig. 3E). Injected brains were stained with the anti-C6S antibody CS56 [42], demonstrating an increased C6S level associated with WFA-stained PNNs (Fig. S7C). In this *AAV-chst3-*injected knockout animals, their 3-h memory was restored to the normal level, demonstrating that the memory deficit of the knockout can be corrected by restoring the level of C6S in the PRh (Fig. 3F).

These results show that the down-regulation of permissive C6S in the PNNs of *chst3* knockouts leads to very premature memory loss.

### Restoration of C6S levels rescues ARMI

**Fig. 4:**
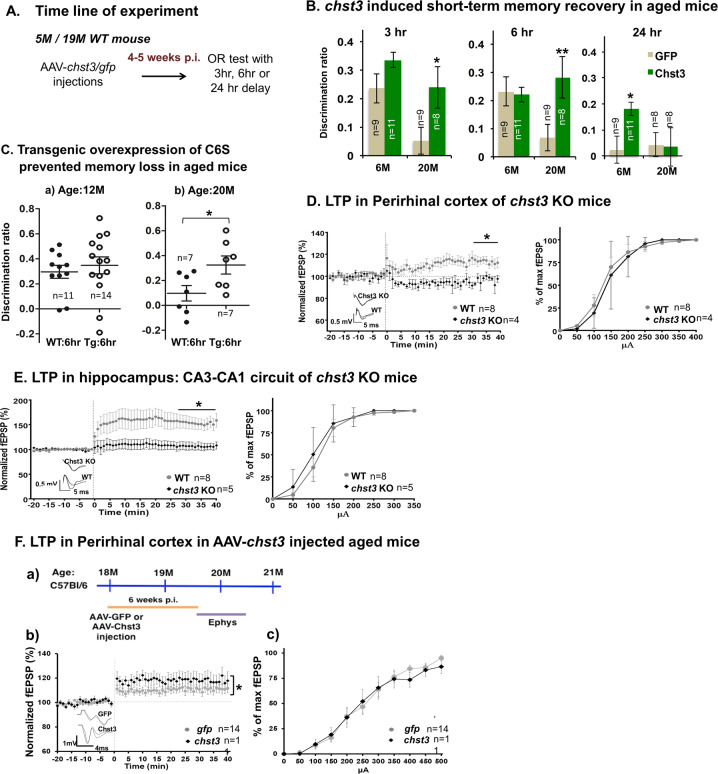
ARMI is prevented in animals overexpressing C6 sulfotransferase. Restoration of C6S restores LTP in brain slices. **A** Experimental timeline of AAV-chst3 injection to 5 M or 19 M C57BL/6 mice. **B** Age-mediated memory loss in C57bl/6 mice and SOR memory restoration by injection of AAV1-*Chst3* to PRh. Three hours memory retention: *p = 0.023. Six hours memory retention: **p = 0.0071. In young mice (6 M), 24 h memory was enhanced after AAV-chst3 injection. *p = 0.0317. Data present as mean ± SEM. All data were analysed by unpaired two-tailed *t*-test. **C** In *chst3* transgenic mice, age-mediated memory loss was absent. Age: 20 M, 6 h memory retention: WT vs Tg *p = 0.0361. Data present as mean ± SEM. **D** LTP in perirhinal cortex slices in *chst3* KO mice. Left: LTP is significantly decreased (two-way RM ANOVA: *p = 0.018). Left inset: examples of fEPSP. Right: I/O curve of evoked fEPSP amplitude (two-way RM ANOVA, n.s. *p* = 0.758). **E** LTP is increased in CA3-CA1 in *chst3* KO mice (two-way RM ANOVA, *p = 0.010). Left inset: Examples of fEPSP. Right: I/O curve (two-way RM ANOVA, n.s. *p* = 0.457). Graphs are mean ± SEM. **F** LTP in perirhinal cortex in AAV-*chst3* injected aged mice. (a) Timeline. (b) LTP in the perirhinal cortex (two-way RM ANOVA: *p = 0.036). Left inset: examples of fEPSP. (c) I/O curve (two-way RM ANOVA, *p* > 0.05).

Having established that low C6S levels can impair memory, we asked whether the low C6S observed in ageing is implicated in memory loss. To do this, we tested whether the expression of *chst3* to reinstate the level of C6S would rescue memory in aged animals. Young (5 M) and aged groups (19 M) of C57BL/6 animals were tested for SOR, demonstrating a profound memory deficit in the aged animals as described above. These elderly animals and young animals were injected with AAV1*-chst3* into PRh. Five weeks later animals were tested for SOR memory at 6 M and 20 M of age (timeline Fig. 4A). In the aged animals, over-expression of chst3 led to a restoration of SOR memory at the 3 and 6 h time points almost to levels normally observed in young animals. In young animals injected with AAV1-*chst3*, 3 and 6 h memory remained normal (Fig. 4B). However, these young 6 M animals injected with AAV1-*chst3* also showed changes very similar to those caused by ChABC injection, with SOR memory persisting for 24 h (Fig. 4B). There was no effect on SA or MB in these injected animals, as expected because only PRh was affected by the virus injections. C6S levels measured by CS56 immunostaining were increased in the injected region (Fig. S7C).

We next investigated the effects of a global increase in C6S. Transgenic mice constitutively overexpressing *chst3*, in which C6S levels in P60 adults is increased from 4 to 20%, have previously been shown to have enhanced plasticity as adults [13]. In contrast to the focal C6S increase caused by an injection of the AAV1-*chst3* virus, these *chst3* over-expressing mice demonstrated increased C6S throughout the brain. We asked whether these C6S overexpressing mice are protected from ARMI. At 20 M these *chst3* overexpressing transgenic mice showed no memory deficit, possessing normal 6 h memory in SOR while the littermate control group showed a memory deficit similar to the aged animals described above (Fig. 4C). Because the *chst3* expression in these transgenic mice is general rather than focal, we also tested SA memory and MB behaviour and found the age-related deficit in MB behaviour was also prevented while SA memory still showed a deficit (Fig. S7D, E).

The physiological effects of ageing and variations in C6S in the PRh and hippocampus were examined by recording LTP and input/output curves in acute slices. In c*hst3* knockout animals, which have low C6S and show premature memory loss, the mice demonstrated a complete loss of LTP in layer II/III following temporal cortex stimulation and a similar loss in the hippocampus: the input/output relation was not changed (Fig. 4D, E). In aged animals, LTP in the cortex was similarly diminished (Fig. 4C). However, an over-expression of *chst3* in the PRh using AAV-*chst3* restored the LTP in the aged mice to the level of young adult (3–6-month-old) animals, but with no change in the input/output curve (Fig. 4F). These results are consistent with an overall higher level of inhibition in animals with low C6S levels, with previous evidence of PNN control of cortical excitability [43], and with previous evidence of increased inhibition in the aged cortex [44, 45]. Together these results show that the level of C6S has a profound effect on memory and synaptic plasticity. Low C6S leads to memory deficits while restoration of C6S levels in aged animals restores memory and plasticity.

### Manipulation of C6S increases inhibitory synapses on PV^+^ interneurons

**Fig. 5:**
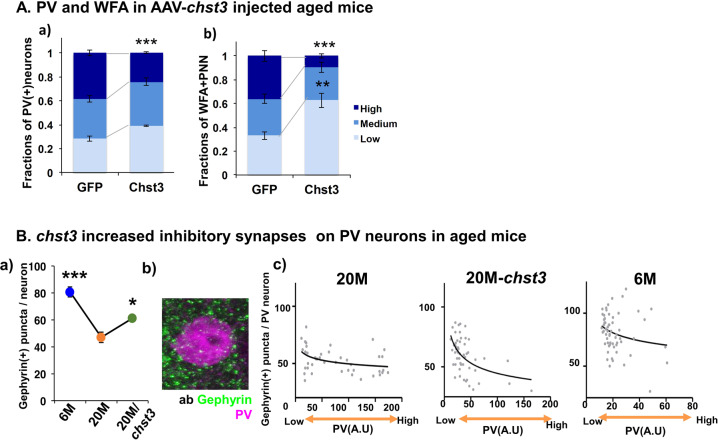
Restoration of C6S affects PV levels and inhibitory synapses. **A** PV and WFA network in AAV-*chst3* injected aged mice (a) PV network. High: ***p = 0.0001. Low: n.s. *p* = 0.079. (b) WFA network. High: ***p < 0.0001. Low: **p = 0.0016. **B** (a), (b) *chst3* increased the inhibitory synapses on PV neurons in aged mice. (a) n = 6/group. 6 M vs 20 M ***p = 0.0002. 20 M vs 20M-*chst3*, *p = 0.01. Unpaired two-tailed *t*-test. (b) Staining profiles of gephyrin (+) synaptic puncta on PV cells. **B** (c) Correlation profiles between numbers of gephyrin synaptic puncta and PV intensity. 20 M: correlation *p = 0.0186, Spearman *r* = −0.3576, 20M-Chst3: Correlation ***p = 0.0003, Spearman *r* = −0.4717, 6 M: Correlation *p = 0.0364, Spearman *r* = −0.2686.

Previous work has shown that during memory acquisition PV levels in PV^+^ interneurons are decreased, probably caused by an increase in the number of inhibitory inputs to PV^+^ cells [26]. Is a similar mechanism driving ARMI? We show above that ageing influenced PV levels, with an increase in the number of PV^+^ interneurons that express high levels of PV (high expressers) and a decrease in those expressing low levels of PV (low expressers). Also in our experiment above, treatment of PRh with ChABC (Fig. 2D) led to a decrease in PV (similar to the result described by Donato et al. [26]), and enhanced SOR memory. We, therefore, asked whether during memory restoration by AAV-*chst3* injections there would be a restoration in the number of inhibitory synapses contacting PV^+^ interneurons. AAV-*chst3* was injected into PRh and after 5 weeks PV levels and synapse numbers were measured by immunostaining and PNNs were assayed by WFA lectin staining. In the AAV-*chst3-*injected PRh, the number of high PV-expressing neurons decreased while the number of low-expressing neurons increased, similar to the effect of ChABC treatment (Fig. 5Aa). Overexpression of *chst3* additionally lowered WFA lectin staining of PNNs as seen previously [46] (Fig. 5Ab). We asked whether the number of inhibitory synapses on PV + interneurons was changed. In aged animals the overall number of gephyrin+ synapses on PV cells was decreased compared to 6 M animals, while treatment with AAV-*chst3* to restore C6S levels and memory led to the restoration of the number of gephyrin synapses towards the level of young animals (Fig. 5Ba, b). Plotting PV levels *versus* the number of gephyrin^+^ synapses for individual neurons revealed a close inverse correlation between the number of gephyrin^+^ synapses on PV^+^ cells and their level of PV expression [41]. The slope of this correlation was greater at younger ages than in aged animals (Fig. 5Bc).

Together, these results suggest a mechanism through which PNNs can influence ARMI. When aged PNNs lose C6S and become more inhibitory, it leads to a decrease in inhibitory inputs to GABAergic PV^+^ neurons, causing increased PV expression and increased cortical inhibition. The increased GABAergic inhibition impairs memory acquisition. Removing PNNs or restoring C6S levels enables increased inhibitory synapses on PV^+^ interneurons, facilitating the restoration of normal memory.

## Discussion

In the current study, we asked whether PNNs and changes in sulphation of their constituent CS-GAGs are involved in the control of memory and plasticity and whether manipulation of their sulphation can restore memory and prevent ARMI. We used three behavioural models that are achievable by very aged mice, which struggle with water and Barnes mazes. In SOR, animals demonstrate by spontaneous exploratory behaviour whether they remember objects as the novel. SOR depends on the PRh, so interventions can be injected focally. SA involves spontaneous exploratory behaviour in which normal animals explore the three arms of a Y-maze in turn, and normally remember not to re-enter the same arm immediately. SA is a spatial task and requires hippocampal function. MB is a species-specific exploratory behaviour in which mice bury exposed glass marbles. MB has been used to test anxiety treatments, but a recent reassessment concluded that MB seems to be a natural exploratory response to a novel environment, rather than being triggered by mood disturbance [35]. It is therefore a test of exploratory behaviour, indicating general cortical function. It involves the hippocampus and frontal and prefrontal cortex [35, 36]. All of these tests showed an age-related deficit. We tested whether PNNs are involved in these deficits. PNN function depends on the CS-GAGs of CSPGs. Focal injection of the CS-GAG-digesting enzyme ChABC to the PRh of aged mice restored SOR but did not affect SA and MB which rely on different brain areas that were not digested. However, an effect of ChABC demonstrates the involvement of CS-GAGs but does not in itself implicate PNNs, because ChABC digests CS-GAGs both in PNNs and in the diffuse ECM in the CNS. To demonstrate a specific role of PNNs and their CS-GAGs in ARMI, transgenic animals with a deletion of *hapln1* (a link protein that stabilizes PNNs) was used. These animals have very attenuated PNNs, but normal overall levels of CSPGs [38]. When these PNN-attenuated animals were allowed to age they showed no ARMI, demonstrating the involvement of PNNs. CNS plasticity, including memory, is strongly influenced by the ECM, particularly by PNNs. They are involved in the closure of critical periods for plasticity [2, 5, 32, 47]. Attenuation or digestion of PNNs restores many forms of plasticity. CSPGs and PNNs have been implicated in several forms of memory. Digestion or transgenic attenuation of PNNs in normal animals prolongs object recognition memory, enables unlearning of stressful memories and addiction and enables social memory [20–22, 48]. CSPG and PNN manipulation can also restore memory lost due to pathology in tauopathy and amyloid-beta models of Alzheimer’s disease, and PNNs are also involved in neuroprotection and other functions in ageing [29–31, 33, 49]. In the current study, digestion did not enhance 24 h memory retention in aged animals while *hapln1* knockout did. This may reflect that ChABC only removes CSs while anchorage of CSPG proteins to PNNs is affected in the knockouts.

The inhibitory function of CSPGs relies mainly on their sulphated CS-GAG chains, with different forms of sulphation having different properties. Overall, 4-sulphated CS-GAGs (C4S) are inhibitory to regeneration and plasticity while C6S is more permissive [12, 13, 17]. The sulphation pattern in the CNS changes at key times, with the ratio of C4S/C6S increasing after embryogenesis and further at the end of critical periods for plasticity [38, 50]. PNN CS-GAG sulphation finally changes with ageing with a significant reduction of C6S, rendering the PNN GAGs more inhibitory [19, 51]. This led to the hypothesis that the increasing ratio of C4S/C6S with ageing, making PNNs more inhibitory, contributes to the development of ARMI. The hypothesis predicts that increasing or decreasing C6S through manipulation of the main CS-6-sulfotransferase *chst3* would have effects on memory and ARMI. Animals with transgenic deletion of *chst3* were tested for the timing of memory loss. These animals showed early impairment in SOR, SA and MB. This implies that permissive CS-GAGs are needed throughout life in order to compensate for age-related deficits that threaten normal memory. In addition, the hypothesis predicts that restoration or preservation of C6S levels should enable better memory in aged animals. We tested this prediction by injecting AAV-*chst3* into the PRh, and testing transgenic animals that globally overexpress *chst3. Chst3* injection into PRh leads to a focal increase in C6S, and this restored SOR (which relies on the PRh) in aged animals. A transgenic animal with a global overexpression of *chst3* was also tested, and this showed a general halt to ARMI, with normal preserved SOR, SA and MB in aged animals. Together these results show that the loss of C6S in PNNs is involved in ARMI, and restoration of C6S levels can halt ARMI and restore memory in aged animals.

What might be the mechanism by with the sulphation of CS-GAGs in PNNs affects memory?

Previous work has shown that memory acquisition and associative motor learning involves an increase in the number of inhibitory synapses on PNN-bearing PV^+^ interneurons, leading to decreased GABA production. Moreover, ChABC digestion both enabled memory and increased synapses on PV^+^ interneurons, and similar changes were seen in the deep cerebellar nucleus in associative motor learning [23, 26, 41, 52]. The implication is that the inhibitory PNNs that appear with ageing might block new synapse formation of PV^+^ interneurons, so inhibiting memory acquisition. The results support this hypothesis because the number of inhibitory synapses on cortical PV^+^ neurons is decreased in aged brains and the PV levels in PV^+^ interneurons are increased. The interventions that restore memory, ChABC and increased *chst3*, both restore inhibitory synapse number on PV^+^ neurons. C6S is permissive to the growth of neuronal processes while C4S is inhibitory, probably due to different levels of signalling through the PTPsigma receptor and interactions with other ECM molecules;[17, 53, 54] this may make it possible for new terminal sprouts to penetrate PNNs that are relatively rich in C6S. The PV levels in PV^+^ interneurons indicate the level of GABA-producing GAD-67 [41] and therefore suggest an age-related increase in overall GABA-mediated cortical inhibition. This pyramidal cell disinhibition may lead to activation of PV^+^ cells feeding back on pyramidal cells causing inhibition. Previous experiments have shown that ChABC treatment modulates the oscillatory behaviour of neural circuits in several brain areas [43, 55].

Ageing has been associated with a deficit in short-term plasticity in the CNS, as measured by a decline or loss of LTP. Physiology of acute cortical slices showed defective LTP in our aged brains, and LTP was restored in animals that had received AAV-*chst3* injections.

How might changes in overall levels of inhibition mediated by PV^+^ GABAergic interneurons affect memory acquisition? A possible scenario comes from investigations of the number and distribution of cells that form a memory engram. Here the strength and sustainability of memory may depend on the number of distributed neurons involved, which in turn would be influenced by overall excitability [56, 57].

There are several probable participants in ARMI as well as PNNs [58–61]. However, PNNs are rich in potential therapeutic targets, including the sulfotransferase enzymes, the production of hyaluronan [62] and the maintenance of PNNs by OTX2 [4]. An anti-C4S antibody has proven successful at memory restoration [30].

Overall the results of this study demonstrate a mechanism for the loss of memory in the aged brain and indicate that treatments targeting PNNs have the potential to ameliorate memory deficits associated with ageing.

## Materials and methods

### Mice

Wild type (WT) C57BL/6J (Charles river, UK) mice were used for the ChABC and AAV-*Chst3* treatment studies. For experiments on C6S and memory, two transgenic mouse models were used; c6st-1 (encoded by chst3 gene) knockout mice [12] and chst3-1 overexpressing Tg mice [13]. Age-matched littermates were used as control mice. Animals had unrestricted access to food and water and were maintained on a 12 h light/dark cycle (lights off at 7:00 p.m.). All experiments were carried out in accordance with the UK Home Office Regulations for the Care and Use of Laboratory Animals and the UK Animals (Scientific Procedures) Act 1986. Group sizes were calculated using power analysis, using previous work on PNNs and memory for effect size and variance.

### Generation of adeno-associated viral vectors

The plasmid encoding AAV-PGK-chst3 was made by amplifying the mouse chst3 sequence from plasmid MR207541 (OriGene) via the primers 5′ GGAATTCATAGGGCGGCCGGGAA 3′ and 5′ AGCGCTGGCCGGCCGTTTAAAC 3′ and was cloned into plasmid AAV-PGK-Cre (Addgene plasmid # 24593) between the AfeI (NEB, R0652) and EcoRI (NEB, R0101) sites to substitute the Cre recombinase gene. The eGFP sequence of AAV-CMV-eGFP (Addgene plasmid # 67634) was amplified using the primers 5′ GGAATTCATGGTGAGCAAGGGCGAG 3′ and 5′ AGCGCTTTACTTGTACAGCTCGTCCATG 3′, which was cloned into the digested AAV-PGK-backbone. These virus vectors were turned into a recombinant adeno-associated viral vector with serotype 1 as described in the previously published protocol [63]. For the present study, the following vectors were produced: AAV1-PGK-*chst3* 1.44 × 10^12^ gc/ml; AAV1-PGK-GFP 1.42 × 10^12^ gc/ml; AAV1-SYN-GFP 8.99 × 10^12^ gc/ml

### Animal surgeries

Animal surgeries were performed under isoflurane anaesthesia. ChABC (50 U/ml in PBS, Seikagaku), or AAV vectors (AAV1-PGK-Chst3 or AAV1-PGK-GFPor AAV1-SYN-GFP) was stereotaxically injected to six different sites in the PRh (1 × 10^8^ particles in total, 3 per hemisphere, 0.5 μl with a speed of 0.2 μl/min). Injections were made with a 10 μl Hamilton syringe and a 33 gauge needle at the following sites (in mm from bregma and the surface of skull): 1. anterior-posterior (AP): −1.8; lateral (L): ± 4.6; ventral (V): −4.4. 2. AP: −2.8; L: ± 4.8; V: −4.3. 3. AP: −3.8; L: ± 4.8; V: −3.8. AAV1-SYN-GFP was injected as a control viral vector for chst3 gene delivery to chst3 KO mice. Animals were randomly assigned to groups, and testing was performed by blinded observers.

### SOR task

The SOR task was performed as previously described for mice [31]. Details in the supplementary information. Data was not used from animals failing to participate in a test.

### SA test

The Y maze was made by three white, opaque Perspex plastic arms (8 cm width, 20 cm length, 35 cm height) at a 120° angle from each other. There were no visual cues inside the maze. Each animal was allowed to freely navigate all three arms for 5 min after placing it at the centre of multiple arms via a tube. The number of arm entries and the number of trials were recorded in order to calculate the percentage of an alternation. An entry occurs when all four limbs are within the arm. The inside of the Y maze was cleaned with 50% ethanol between trials and allowed to dry.

### MB test

Standard polycarbonate rat cages (26 cm × 48 cm × 20 cm) with fitted filter-top covers was used as a testing chamber and fresh, unscented mouse bedding material to each cage to a depth of 5 cm. Glass toy marbles (assorted styles and colours, 15 mm diameter, 5.2 g in weight) were placed gently on the surface of the bedding in 3 rows of 5 marbles. Each animal was carefully placed into a corner of the cage containing marbles, as far from marbles as possible, and the lid was placed on the cage and remained for 30 min. Marble, as buried if two-thirds of its surface area is covered by bedding, was counted by a scorer blind to the genotype of the mouse.

### Diaminobenzidine (DAB) staining

Details in the Supplementary information.

### Fluorescent staining/analysis

Sections were blocked with 5 % normal goat serum (NGS) or normal horse serum (NHS) in PBS-T for 1 h at RT. The primary antibodies (PV; 1:1000, Swant CS56; 1:100, Sigma-Aldrich; Biotin-WFA; 1:100, Sigma-Aldrich, Gephyrin; 1:200, Synaptic system) were incubated overnight at 4 °C. Following three washes in PBS they were incubated for 2 h at RT with the appropriate secondary antibody conjugated with Alexa fluor 647, Alexa fluor 488 or Alexa fluor 568 or Streptavidin-Alexa fluor 647 (Molecular Probes, Invitrogen) diluted 1:500 in PBS-T. incubated with secondary antibodies for 2 h. Sections were rinsed and mounted on 1% gelatin-coated slides with FluorSave™ Reagent (Merck Millipore, Germany).

For synaptic puncta, quantification images were captured using a Leica SPE confocal microscope using ×63 objectives with a 1024 × 1024 image resolution (*n* = 6 per group). At least 3 z-stack images (total 5 µm) were taken per section with at least three sections analyzed per animal (~360 µm apart). Images contained at least 5 PV positive neurons. At least 50 PV^+^ neurons per animal were analysed for gephyrin (+) puncta quantification. Synaptic puncta analysis was performed with an automated custom script using an imageJ 1.29 plugin (available from c.eroglu@cellbio.duke.edu) [64].

### Electrophysiology

Animals were anaesthetized with an overdose of isoflurane, killed by cervical dislocation and decapitated. The brain was rapidly removed and placed in ice-cold cutting solution bubbled with 99% O_2_ containing the following (in mM): 126 NaCl, 2.5 KCl, 1 CaCl_2_·H2O, 2 MgCl_2_, 1.25 NaH_2_Po_4_·H_2_O, 10 NaHCO_3_, 5 D-glucose, 0.4 ascorbic acids, 3 myoinositol, 3 pyruvate, and 15 HEPES, adjusted to pH 7.35. For the cutting and the recording of the perirhinal cortex slices from the 20 M aged animal, we used a modified ACSF continuously oxygenated (carbogen: 95% O_2_-5% CO_2_) and containing the following (in mM): 124 NaCl, 3 KCl, 1.5 CaCl_2_·2H_2_O, 1.5 MgCl_2_ 6H_2_O, 1.25 NaH_2_Po_4_·H_2_O, 26 NaHCO_3_, 10 D-glucose, 0.01 Glycine, 1 L-ascorbic acid, and 2 Na Pyruvate, adjusted to pH 7.35. Vibratome slices (380 µμm) were placed into interface chambers superfused with artificial CSF. Evoked field EPSPs (fEPSP) for the perirhinal cortex were recorded from layers II/III. fEPSP for the hippocampus were recorder recorded placing the stimulation electrode in the stratum radiatum of the CA3 field and the recording electrode in the same layer of the CA1 field. For LTP induction,4 bursts were delivered at an interval of 15 s, each composed of ten trains at an interval of 0.2 s, each composed by 4 pulses at an interval of 10 ms and 0.2 ms of duration with 3 V of amplitude. Subsequently, fEPSPs elicited by stimulations at an interval of 20 s were recorded for a further 40 min.

Further details in the Supplementary information.

## Supplementary information

Supplemental Material
