# Supplementary material for: Chondroitin 6-sulphate is required for neuroplasticity and memory in ageing
Source: Mol Psychiatry. 2021 Jul 16;26(10):5658–68. doi: 10.1038/s41380-021-01208-9 (PMC8758471; doi:10.1038/s41380-021-01208-9)
Supplement: Supplementary file 1 — Supplemental Material [file 41380_2021_1208_MOESM1_ESM.docx]

**Chondroitin 6-sulphate is required for neuroplasticity and memory in ageing**

Yang, SJ et al. Supplementary Methods and Figures.

MATERIALS and METHODS

Mice

The normal wild type (WT) C57BL/6J (Charles river, UK) mice were used for ChABC treatment study and 6M of age represents for young adults and 20M for aged group. In order to test between C6S and memory, two transgenic mouse models were used; c6st-1 (encoded by chst3 gene) knockout mice (21) and chst3-1 overexpressing Tg mice (22). Age-matched littermates were used as control mice. Animals had unrestricted access to food and water, and were maintained on a 12 hr light/dark cycle (lights off at 7:00 P.M.). All experiments were carried out in accordance with the UK Home Office Regulations for the Care and Use of Laboratory Animals and the UK Animals (Scientific Procedures) Act 1986. Group sizes were calculated using power analysis, using previous work on PNNs and memory for effect size and variance.

Generation of adeno-associated viral vectors

The plasmid encoding AAV-PGK-chst3 was made by amplifying the mouse chst3 sequence from plasmid MR207541 (OriGene) via the primers 5’ GGAATTCATAGGGCGGCCGGGAA 3’ and 5’ AGCGCTGGCCGGCCGTTTAAAC 3’ and was cloned into plasmid AAV-PGK-Cre (Addgene plasmid # 24593) between the AfeI (NEB, R0652) and EcoRI (NEB, R0101) sites to substitute the Cre recombinase gene. The eGFP sequence of AAV-CMV-eGFP (Addgene plasmid # 67634) was amplified by using the primers 5’ GGAATTCATGGTGAGCAAGGGCGAG 3’ and 5’ AGCGCTTTACTTGTACAGCTCGTCCATG 3’, which was next cloned into the digested AAV-PGK-backbone. These virus vectors were turned into a recombinant adeno-associated viral vector with serotype 1 as described in previously published protocol (J. Verhaagen *et al.*, 2018). For the present study, the following vectors were produced: AAV1-PGK-*chst3* 1.44x10^12^ gc/ml; AAV1-PGK-GFP 1.42x10^12^ gc/ml; AAV1-SYN-GFP 8.99x10^12^ gc/ml

J. Verhaagen *et al.*, Small Scale Production of Recombinant Adeno-Associated Viral Vectors for Gene Delivery to the Nervous System. Methods Mol. Biol. 1715, 3–17 (2018).

Animal surgeries

Animal surgeries were performed under isoflurane anesthesia. ChABC (50 U/ml in PBS, Seikagaku), or AAV vectors (AAV1-PGK-Chst3 or AAV1-PGK-GFPor AAV1-SYN-GFP) was stereotaxically injected to six different sites in the PRh (1x10^8^ particles in total, 3 per hemisphere, 0.5 μl with a speed of 0.2 μl/min). Injections were made with a 10 μl Hamilton syringe and a 33 gauge needle at the following sites (in mm from bregma and the surface of skull): 1. anteriorposterior (AP): -1.8; lateral (L): ± 4.6; ventral (V): -4.4. 2. AP: -2.8; L: ± 4.8; V: -4.3. 3. AP: -3.8; L: ± 4.8; V: -3.8. The needle remained in place at the injection site for 3 min before being slowly withdrawn over 2 min. AAV1-SYN-GFP was injected as a control viral vector for chst3 gene delivery to chst3 KO mice. Animals were randomly assigned to groups, and testing was performed by blinded observers.

Spontaneous Object Recognition task

The spontaneous object recognition (SOR) task was performed as previously described for mice (Yang et al, 2015). Briefly, all mice were habituated in three consecutive daily sessions in the empty Y-maze apparatus for 5 min after recovery from surgery. Each test session consisted of a sample phase and a choice phase. In the sample phase, the animal was placed in the start arm and left to explore two identical objects, which were placed on the end of two arms for 5 min. The choice phase followed after a delay of either 1 min, 3 hr, 6 hr as a short-term memory paradigm or 24 hr and 48 hr as a long-term memory paradigm, which the animal spent in the home cage. The choice phase was procedurally identical to the sample phase, except that one arm contained a novel object, whereas the other arm contained a copy of the repeated object. When the animals were assessed, a different object pair was used for each session for a given animal, and the order of exposure to object pairs were counterbalanced within and across groups. All the sessions were recorded by a technician (SH) who is blind to the genotype. The object exploration time was assessed from video recordings of the sample and choice phase by scorer (SY) blind to the genotype of the mouse. The direct nasal or head contacts only were regarded as an exploratory behaviour. A discrimination ratio was calculated by dividing the difference in exploration of the novel and familiar objects by the total object exploration time. Therefore, the discrimination ratio varies from 0 (equal exploration for novel and familiar objects) to 1 (exploration of the novel object only). The test sessions were separated by a minimum of 48 hr. One-way analysis of variance (ANOVA) followed by Tukey post hoc test was used for comparisons of multiple groups with a significance level of p < 0.05, using GraphPad Prism version 5.0. An unpaired two-tailed t-test was used for two-group comparisons.

The minimum sample size per animal experiment required to have meaningful results was 14 animals, which was calculated based on raw data of chABC or control treated 20M old mice using following parameters; type 1 error rate (a):0.05 and power: 0.8 , mu1 -0.002, mu 2 0.3301, sigma 0.214 (http://www.stat.ubc.ca/~rollin/stats/ssize/n2.html).

For some experiments two cohorts of mice were recruited and the results were analysed at the end of experiments.

Spontaneous alternation test

The Y maze was made by three white, opaque Perspex plastic arms (8 cm width, 20 cm length, 35 cm height) at a 120° angle from each other. There were no visual cues inside the maze. Each animal was allowed to freely navigate all three arms for 5 min after placing it at the centre of multiple arms via a tube. The number of arm entries and the number of trials were recorded in order to calculate the percentage of alternation. An entry occurs when all four limbs are within the arm. The inside of Y maze was cleaned with 50% ethanol between trials and allowed to dry.

Marble burying test

Standard polycarbonate rat cages (26 cm x 48 cm x 20 cm) with fitted filter-top covers was used as a testing chamber and fresh, unscented mouse bedding material to each cage to a depth of 5 cm. Glass toy marbles (assorted styles and colors, 15 mm diameter, 5.2 g in weight) were placed gently on the surface of the bedding in 3 rows of 5 marbles. Each animal was carefully placed into a corner of the cage containing marbles, as far from marbles as possible, and the lid was placed on the cage and remained for 30 min. Marble as buried if two-thirds of its surface area is covered by bedding was counted by a scorer blind to the genotype of the mouse.

Immunohistochemistry

Diaminobenzidine (DAB) staining

Sample preparations and the general procedures of immunostaining have been described previously (Yang *et al.*, 2015). In general, 30 µm free floating sections were incubated with 10 % methanol, 3 % H_2_O_2_ in PBS for 20 min at room temperature for quenching endogenous peroxidase activity. Sections were rinsed three times in 0.2 % Triton-X in PBS (PBS-T) and were subsequently blocked with 5 % normal goat serum (NGS) or normal horse serum (NHS) in PBS-T for 1 hr at room temperature. The primary antibodies (PV; 1:1000, Swant; Biotin-WFA; 1:100, Sigma-Aldrich) were incubated over night at 4 °C following rinsing tissues for 5 min three times in PBS-T (0.2 % Triton-X in PBS). Following 3 washes in PBS-T they were incubated for 2 hr at RT with the appropriate biotinylated secondary antibody (Vector Laboratories) diluted 1:500 in PBS-T. Subsequently, sections were incubated with an avidin-biotin system (Vectastain; Vector Laboratories) for 1 hr at room temperature and washed with PBS-T. The immunostaining was visualized with DAB with 3 % H_2_O_2_ (DAB kit; Vector Laboratories) for 1-5 min at room temperature. The sections were mounted on gelatin-coated slides and air-dried. Following dehydrating tissue sections in ascending concentration of alcohols, they are cleared in xylene and coverslipped with DPX. The tissue sections were examined using a light microscope and photographed using a digital camera.

Fluorescent staining/Analysis

Sections were blocked with 5 % normal goat serum (NGS) or normal horse serum (NHS) in PBS-T for 1 hr at RT. The primary antibodies (PV; 1:1000, Swant CS56; 1:100, Sigma-Aldrich; Biotin-WFA; 1:100, Sigma-Aldrich, Gephyrin; 1:200, Synaptic system) were incubated overnight at 4 °C. Following 3 washes in PBS they were incubated for 2 hr at RT with the appropriate secondary antibody conjugated with Alexa fluor 647, Alexa fluor 488 or Alexa fluor 568 or Streptavidin-Alexa fluor 647 (Molecular Probes, Invitrogen) diluted 1:500 in PBS-T then, incubated with secondary antibodies for 2 hours. Sections were rinsed and mounted on 1% gelatin coated slides with FluorSave™ Reagent (Merck Millipore, Germany).

Immunohistochemistry and image capture of each experimental group were performed at the same time using the same settings.

For PV intensity analysis, images were taken from at least 3 sections (approx. 360 µm apart) between bregma -1.8 mm and bregma -3.8 mm using a fluorescent microscope (Leica DM6000) with x20 objectives (n=4 per group). The total PV+ neurons were identified from the whole region of PRh and the intensity of each neuron was measured using ImageJ software and quantified in arbitrary units (A.U). The threshold for the low intensity class was decided at 66% of mean intensity of control group. In the experiment of ChABC treatment to 6M and 20M WT animals, the threshold was set accordingly by the mean PV intensity of 6M Pnase treated control samples. PV neurons were classified into three subclasses as follows: low PV, 0-700 A.U; medium PV, 700-1400 A.U; High PV, >1400 A.U. The ratio of each PV neuronal group against total number of PV neurons was analysed and plotted. In the experiment of AAV-*chst3* injection to aged mice, tissues sections were co-stained with PV antibody and WFA (n=6 per group). The threshold for the low PV and WFA intensity was determined at 66% level of mean intensity of total PV or WFA^+^ neurons from PRh of AAV-GFP injected animals. PV and WFA^+^ neurons were classified into three subclasses as follows: low PV, 0-400 A.U; medium PV, 400-800 A.U; High PV, >800 A.U; low WFA, 0-600 A.U; medium WFA, 600-1200 A.U; High WFA, >1200 A.U.

For synaptic puncta quantification images were captured using a Leica SPE confocal microscope using x63 objectives with a 1024 x 1024 image resolution (n=6 per group). At least 3 z-stack images (total 5 µm) were taken per section with at least 3 sections analyzed per animal (approx. 360 µm apart). Images contained at least 5 PV positive neurons. At least 50 PV^+^ neurons per animal were analysed for gephyrin (+) puncta quantification. Synaptic puncta analysis was performed with an automated custom script using an ImageJ 1.29 plugin (available from c.eroglu@cellbio.duke.edu).

Stereology

For stereological counting of neurons and PNNs, brain sections were stained with NeuN and WFA. Stereology was performed using StereoInvestigator (MBF Bioscience). A total number of 5-6 sections per animal, taken every 12 th brain sections were counted. The whole brain section was first highlighted at 4x objective and PRh contour was outlined. Sample counting windows were identified using the computer software. The number of sample counting windows was normally between 15-20. The number of NeuN or WFA-positive cells in each window was counted at 40x magnification. The counting frame and the counting grid were set by the operator; 40 µm x 40 µm and 200 µm x 200 µm, respectively. Movements among counting frames of each grid were controlled by a motorized x-y microscope stage. Cells located inside the counting frame or touching its top or right borders only were counted. For the Z calibration, the tissue thickness at every counting frame was determined and the mean of sections thickness was taken into account for calculating the total number of cells. The total number of NeuN and WFA-positive cells was calculated using the following equation:


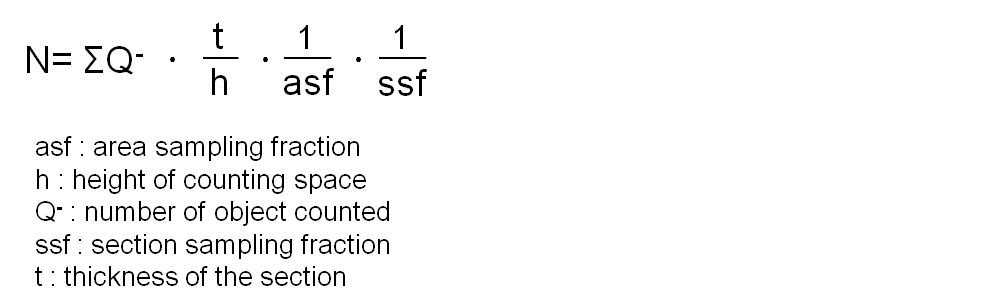


GAG extraction

Biochemical analyses were performed on 5 animals at each age. Sequential extraction of GAGs from rat brains was performed according to the protocol from (Kwok et al. 2015). Briefly, fresh frozen brains were first homogenized sequentially in 4 buffers: buffer 1 (B1: 50 mM Tris-buffered saline (TBS), 2 mM ethylenediamine tetraacetic acid (EDTA)), buffer 2 (B2: 0.5% Triton X-100 in B1), buffer 3 (B3: 1 M sodium chloride in B2) and buffer 4 (B4: 6 M urea in B2). All buffers were supplemented with protease inhibitors. The homogenate was centrifuged at 23,000 xg for 20 min at 4°C after each homogenization. Supernatant was collected and the pellet was homogenized in the next buffer. Supernatants from each buffer were dialyzed in 25 mM Tris-HCl and 5 mM EDTA pH 8.0 overnight at 4°C and subsequently digested with 200 mg/ml pronase (Roche). Peptides were precipitated with 5% trichloroacetic acid (TCA) on ice, centrifuged and the supernatants were collected. Supernatants were then washed four times in 1:1 diethyl ether which was then aspirated. The samples, which contained the glycosaminoglycans (GAGs) were neutralized to pH 7.0 and precipitated with sodium acetate. GAGs were recovered from the pellets after centrifugation, air dried and resuspended in deionized water.

Recovered GAGs were quantified using cetylpyridinium chloride (CPC) turbidimetry assay. Chondroitin sulphate-A (Sigma Aldrich) was used to set up the standard curves.

Fluorophore-assisted carbohydrate electrophoresis (FACE)

Recovered GAGs were digested into disaccharides with 0.1 U chABC (Sigma), then precipitated in EtOH for 16 h at 4°C. For C6S quantification, the samples were further treated further with 50 mU of chondro-6-sulfatase (Seikagaku). Samples were centrifuged, supernatants were collected and dried in a SpeedVac. The dried pellets were derivatized with 2-aminoacridone (AMAC) in sodium cyanoborohydride containing buffer. 0.5 μg of AMAC-conjugated disaccharide samples and standard disaccharides (hyaluronic acid – HA, non-sulfated chondroitin - C0S, C4S, C6S; Seikagaku) were separated on a 30% polyacrylamide gel in Tris Glycine buffer for 30-40 min.

For C4S, gels were imaged in a UV chamber (UVitec). Bands size and intensity were quantified using ImageJ^TM^ software. The intensity of the sample was normalized against the intensity of non-sulphated CS (C0S) and the quantity of disaccharides was calculated using the standard curve of HA electrophoresed in the same gel.

Electrophysiology

Animals were anesthetized with an overdose of isoflurane, humanely killed by cervical dislocation and decapitated. The brain was rapidly removed and placed in ice-cold cutting solution bubbled with 99% O2 containing the following (in mM): 126 NaCl, 2.5 KCl, 1 CaCl2·H2O, 2 MgCl2, 1.25 NaH2Po4·H2O, 10 NaHCO3, 5 D-glucose, 0.4 ascorbic acid, 3 myoinositol, 3 pyruvate, and 15 HEPES, adjusted to pH 7.35. For the cutting and the recording of the perirhinal cortex slices from the 20M aged animal, we used a modified ACSF continuously oxygenated (carbogen: 95% O2-5% CO2) and containing the following (in mM): 124 NaCl, 3 KCl, 1.5 CaCl2·2H2O, 1.5 MgCl2 6H2O, 1.25 NaH2Po4·H2O, 26 NaHCO3, 10 D-glucose, 0.01 Glycine, 1 L-ascorbic acid, and 2 sodium pyruvate, adjusted to pH 7.35. For perirhinal cortex a midsagittal section was made and the rostral part of one hemisphere was cut at 45° to the dorsoventral axis (Cho *et al.*, 2000). The cerebellum was removed from the brain with a further caudal coronal cut. The hemisphere was glued by its rostral end to a Vibratome stage (VT 1000S; Leica). Slices (380 µμm) of perirhinal cortex were taken in the region -2.5mm to -4mm rostral from bregma. For hippocampus the whole brain was glued by its caudal end and coronal sections were taken in the region of dorsal hippocampus. Slices were stored submerged in bubbled, artificial ACSF (20-25°C, same composition as cutting solution, except 2 mM CaCl2, 1 mM MgCl2 and without: ascorbic acid, myoinositol, pyruvate) for 2 hr before the onset of recordings. A single slice was placed in an interface recording chamber superfused by artificial CSF (30°C, flow rate 2 ml/min). Evoked field EPSPs (fEPSP) for perirhinal cortex were recorded from layers II/III from directly below the rhinal sulcus (area 35). A stimulation electrode was placed in layer II/III on the entorhinal side (0.5 mm, area 36) of the recording electrode (Cho *et al.*, 2000). fEPSP for hippocampus were recorder recorded placing the stimulation electrode in the stratum radiatum of the CA3 field and the recording electrode in the same layer of the CA1 field. Stimuli (0.1 ms duration) were delivered to the stimulation electrode at 0.1 Hz. Input/output curves were produced with stimulation intensities from 50 to 450 500 A in steps of 50 A. For monitoring baseline synaptic transmission before LTP induction, fEPSPs were reduced to 40-50 % of the maximum amplitude and recorded for at least 20 min or until responses were stable (< 20 % amplitude change over 30 min). For LTP induction, the following protocol was used: 4 bursts at interval of 15 s, each composed by 10 trains at interval of 0.2 s, each composed by 4 pulses at interval of 10 ms and of 0.2 ms of duration with 3 V of amplitude. Subsequently, fEPSPs elicited by stimulations at interval of 20 s were recorded for further 40 min. Field potentials were amplified with a CyberAmp 320 (Molecular Devices) or an Axoclamp-2B (Axon Instrument), and recorded and analyzed with pClamp (Molecular Devices) or a custom-made software written in LabView (National Instruments). For offline LTP analysis, fEPSPs were averaged across 1 min and the peak amplitude of the mean fEPSP was expressed relative to the preconditioning baseline. The significance of group means was established using repeated-measures (RM) ANOVA.

**
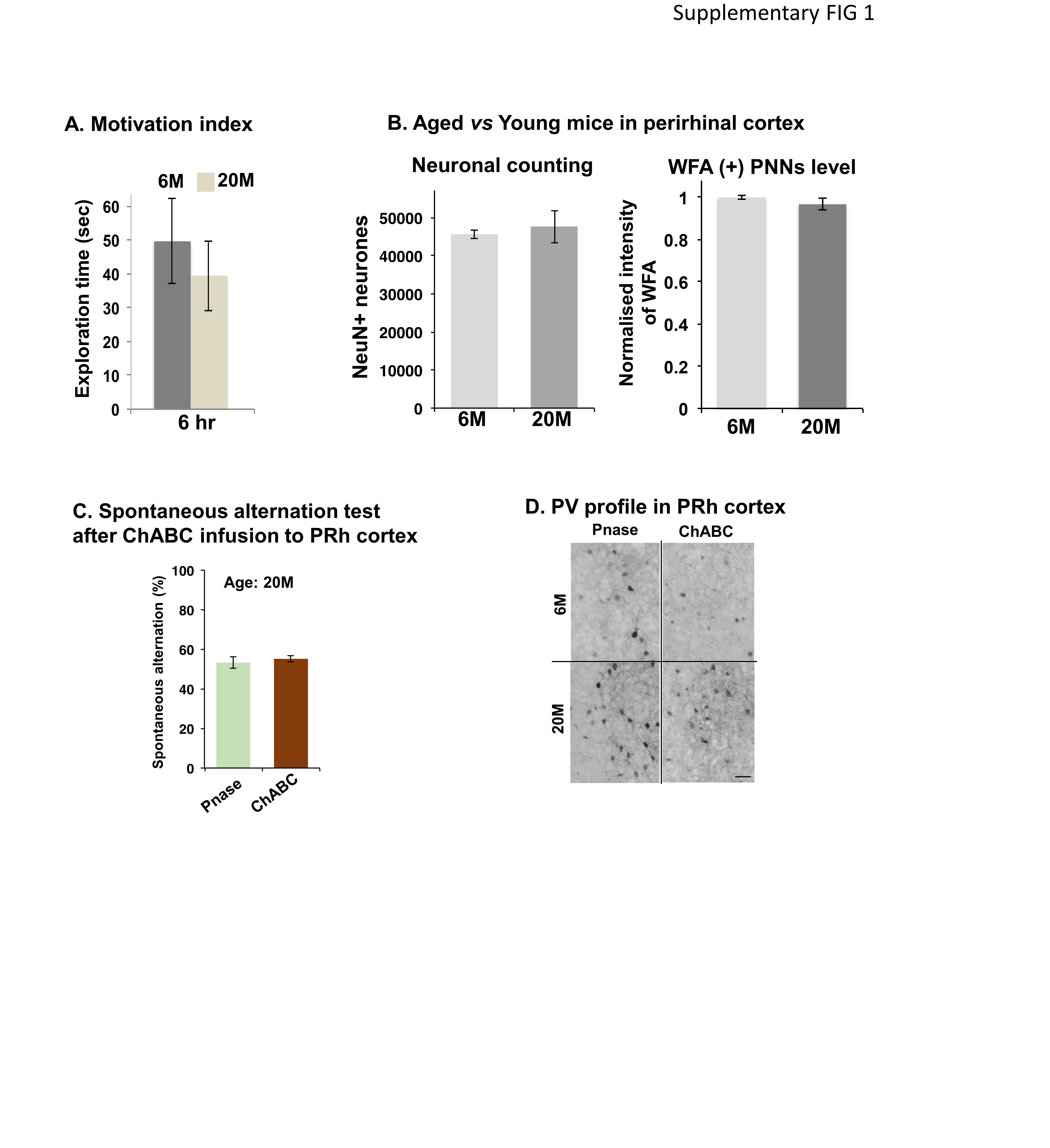
Supplementary Fig. S1** (A) Motivation index indicated by exploration time during sample phase of SOR test. No significant alteration in motivation was displayed between young and aged mice groups. 6M n=4, 20M n=6. (B) Neuronal counting (left) and WFA(+) PNNs level (right) in the perirhinal cortex (PRh) of 6M or 20M old C57BL/6 mice. NeuN(+) n=6/group, WFA(+) n=4/group. (C) Spontaneous alternation performance after ChABC treatment to the PRh in aged mice. Pnase n=8 ChABC n=8. (D) Immunohistochemistry of PV in PRh cortex after ChABC or Pnase injection. Scale bar: 50 µm


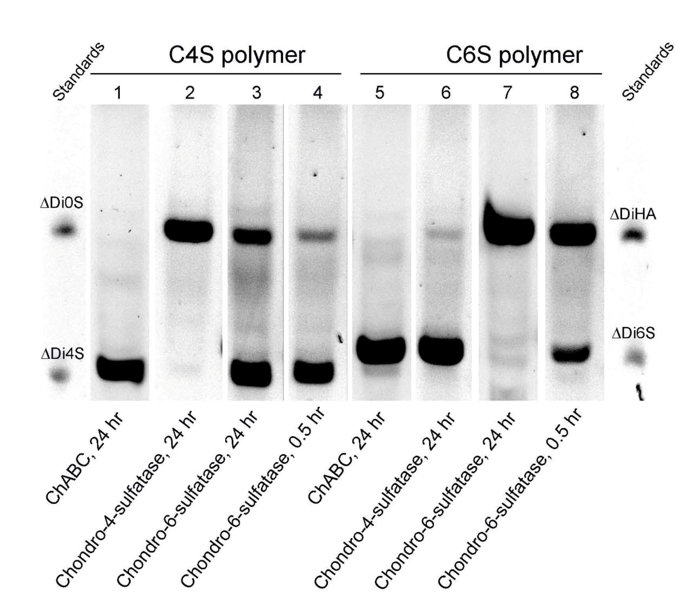


**Supplementary Figure S2 S6.** Digestion of C4S and C6S glycan polymers by chondroitinase and sulfatases. Lanes 1 and 5 show chondroitinase digestion. The CS polymers resolve into a single dense band in each case, containing completely digested C4S and C6S disaccharides. This shows that chondroitinase ABC can completely digest both C4S and C6S (lane 2 and 5). The other lanes show the efficacy of 4 and 6 sulfatases which were not used in this paper.


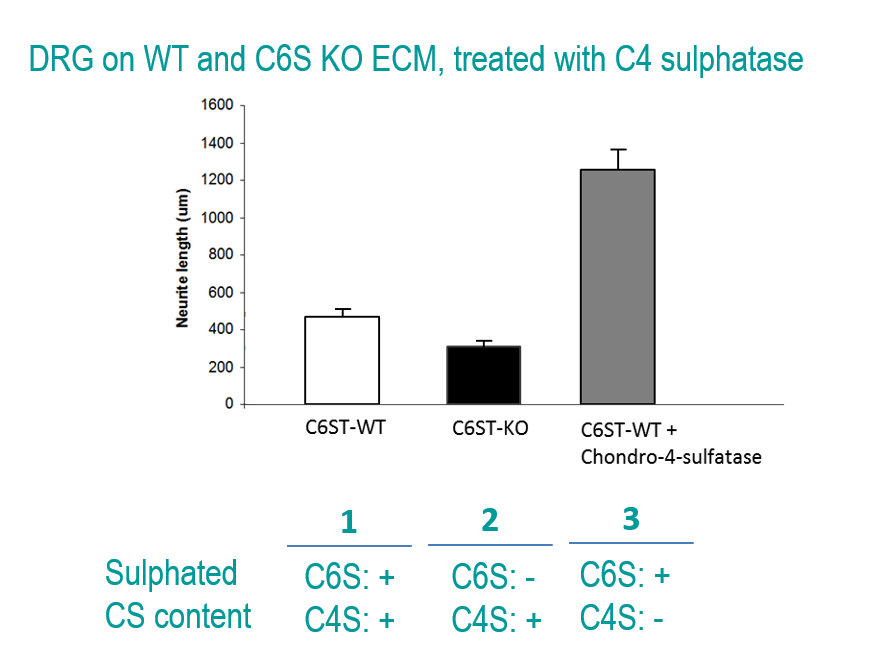


**Supplementary Figure S3 S5.** Sensory axon growth on brain extraellular matrix. On wild-type matrix (left bar) axons grow moderately. Matrix from the C6ST knockout (middle bar) lacks C6S; the matrix contains mainly inhibitory C4S. This C6S knockout matrix is more inhibitory than wild type. In the right hand bar the wild type matrix has been digested with chondro-4 sulfatase to digest away most of the inhibitory C4S, the C6S remaining at normal level. This renders the matrix much more permissive to axon growth.


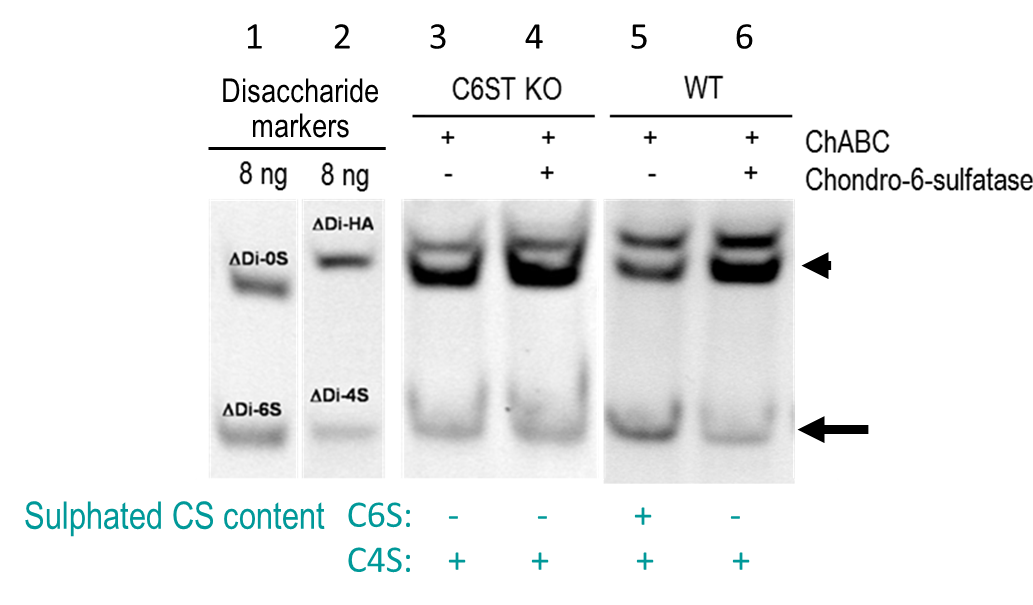


**Supplementary Figure S4 S4.** This FACE gel shows disaccharides released from chondroitin sulphates by digestion with chondroitinase ABC. Lanes 1 and 2 show the migration distance of standard disaccharides, including ΔDi-0S, ΔDi-4S, ΔDi-6S and ΔDi-HA. GAGs were isolated from C6ST KO and WT mice, labelled and electrophoresed to reveal the content of these sulphation forms. In wild-type animals (lanes 5 and 6), digestion with chondro-6-sulfatase leads to a decrease in density of the lower band (arrow) and an increase in the upper band (arrowhead) due to digestion of C6S to non-sulphated C0S. Lanes 3 and 4 show the mono-sulphated CS contenct in C6ST KO. The results show a single band of 4 sulphated disaccharide. The band does not shift or change in density after digestion with chondro-6 sulfatase, indicating that there is no C6S in the proteoglycans from the knockout.

**Supplementary Figure S5 S7.** Length of PNNs on the dendrites of PV^+^ neurons. Sections of 3 month wild-type and *chst3* knockout cortex were stained for PV and WFA. The length of the segments of WFA-stained PNN along the dendrites was measured. There was no significant difference between the groups. Neurons were skeletonised using image J then PNNs quantifed by pixel count (3 animals per group, 15 neurons per animal analyzed).


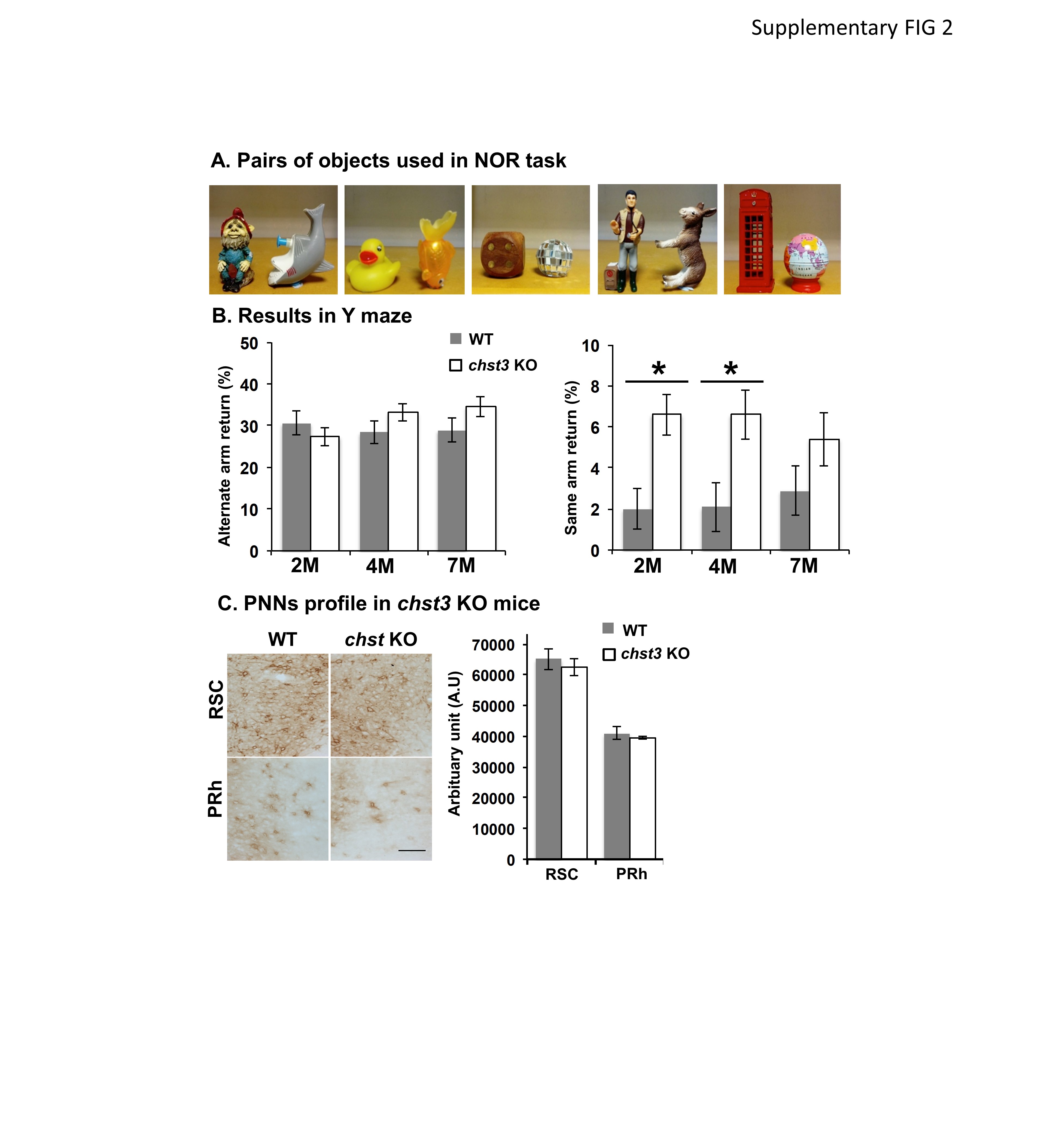


**Supplementary Figure S6 S2** (A) Pairs of objects used in SOR task. (B) Y-maze based spontaneous alternation test in *chst3* KO mice at different ages, 2M: WT n=8, *chst3* KO n=16, 4M: WT n=9, *chst3* KO n=11, 7M: WT n=12, *chst3* KO n=12. Left: alternate arm return. Right: same arm return. Unpaired two-tailed *t*-test 2M: *p=0.018 4M: *p=0.019, 7M: n.s. p=0.2. (C) Analysis of PNNs in *chst3* KO mice, Left: immunohistochemistry of WFA(+) profile in retrosplenial cortex (RSC) and perirhinal cortex (PRh). Scale bar: 50 µm. Right: WFA intensity comparison between WT and *chst3* KO mice. There is no significant difference between WT and *chst3* KO. n=6/group. Data represent as mean ± SEM.

**
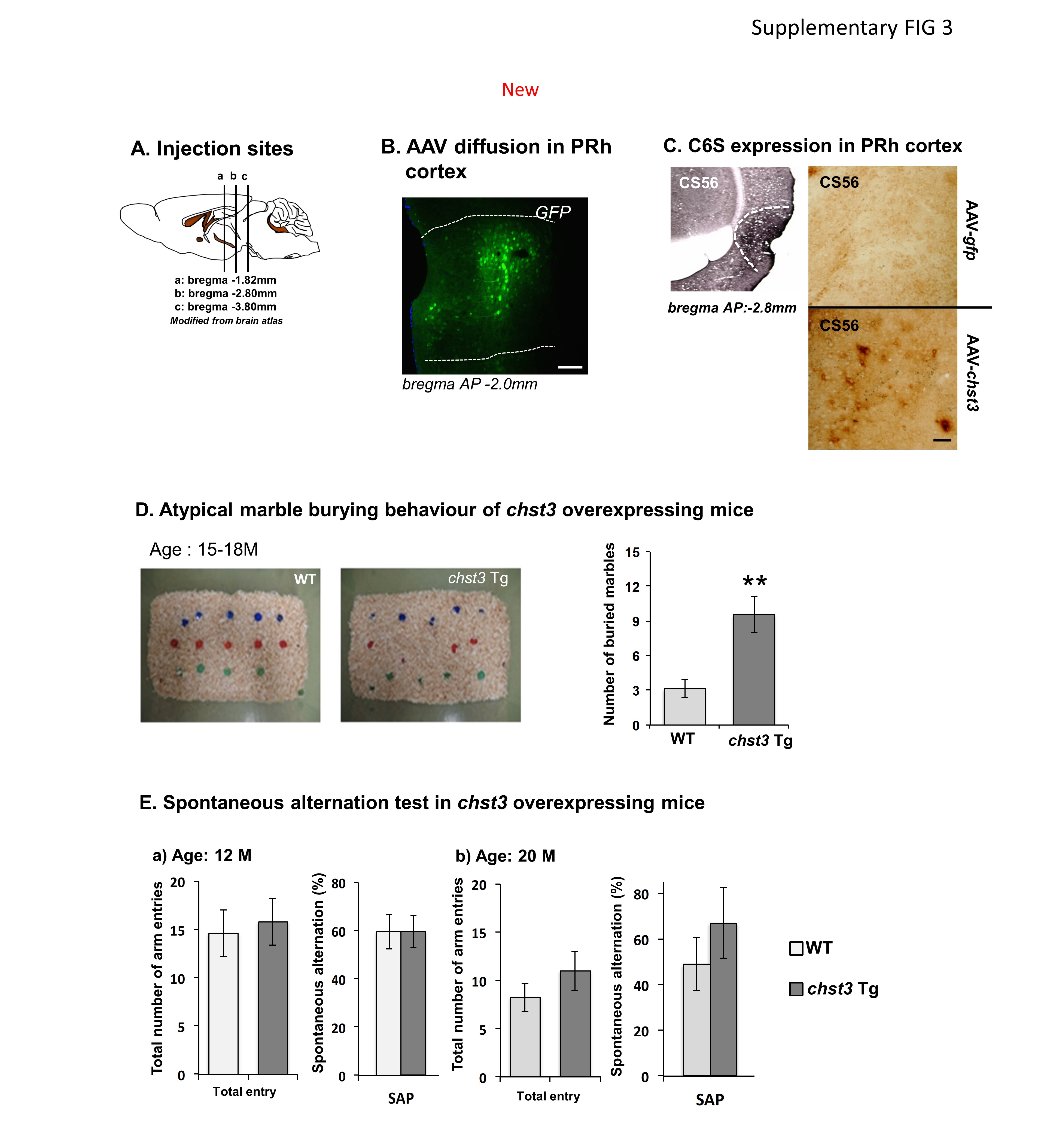
**

**Supplementary Figure S7 S3** (A) Stereotaxic injection sites for the PRh. a: bregma -1.82 mm, b: bregma -2.80 mm, c: bregma -3.80 mm. (B) The profile of AAV-*GFP* diffusion in the PRh. Scale bar: 100 µm. (C) C6S expression induced by AAV-*chst3* was detected by CS56 antibody in C57BL/6 wild type (WT) mice at 20M. AAV-*GFP* injection has no CS56 positive staining. Scale bar: 50 µm (D) Atypical marble burying behaviour of *chst3* overexpressing transgenic mice at the age of 15-18M. Left: representative images of marble burying test. Right: number of buried marbles. WT n=8, *chst3* Tg n=9. Unpaired two-tailed *t*-test **p=0.0038. Data represent as mean ± SEM. (E) Spontaneous alternation test in *chst3* overexpressing Tg mice. Age: 12M, WT n=5, *chst3* Tg n=9. Age: 20M, WT n=4, *chst3* Tg n=4. There is no difference between WT and *chst3* Tg mice at 12M and 20M.
